# A Randomized-Controlled Trial of EMDR Flash Technique on Traumatic Symptoms, Depression, Anxiety, Stress, and Life of Quality With Individuals Who Have Experienced a Traffic Accident

**DOI:** 10.3389/fpsyg.2022.845481

**Published:** 2022-03-24

**Authors:** Alişan Burak Yaşar, Emre Konuk, Önder Kavakçı, Ersin Uygun, İbrahim Gündoğmuş, Afra Selma Taygar, Esra Uludağ

**Affiliations:** ^1^Department of Clinical Psychology, İstanbul Gelişim University, İstanbul, Turkey; ^2^Davranış Bilimleri Institute, İstanbul, Turkey; ^3^Department of Psychology, İstanbul Kültür University, İstanbul, Turkey; ^4^Department of Psychology, İstanbul Bilgi University, İstanbul, Turkey; ^5^Department of Psychiatry, Kırıkkale Yüksek İhtisas Hospital, Kırıkkale, Turkey; ^6^Department of Psychology, Üsküdar University, İstanbul, Turkey; ^7^Department of Psychology, Sabahattin Zaim University, İstanbul, Turkey

**Keywords:** EMDR, flash technique, mhGAP, randomized-controlled study, traumatic stress

## Abstract

The Flash Technique of Eye Movement Desensitization and Reprocessing (EMDR) is widely recognized for its effectiveness in reducing the effects of emotional responses associated with traumatic memories. Using a randomized-controlled trial methodology, this study attempts to establish the efficacy of the EMDR Flash Technique. This study’s sample includes volunteers who were involved in traffic accidents and were given the randomized EMDR Flash Technique and Improving Mental Health Training for Primary Care Residents (mhGAP) Stress management module. The participants were given a socio-demographic data form, the Depression-Anxiety-Stress 21 scale (DASS-21), the Impact of Event Scale-Revised (IES-R), and the WHOQOL Quality of Life scale. Participants were evaluated using measurements taken before and after the application, as well as a one-month follow-up. The mean age of the participants was 36.20 (11.41) years and 82.1% (*n* = 32) were female. The DASS-21 Anxiety (*η*^2^ = 0.085), IES-R Intrusion (*η*^2^ = 0.101), Avoidance (*η*^2^ = 0.124), Total (*η*^2^ = 0.147), and WHOQOL-BREF Psychological (*η*^2^ = 0.106) score improvements of the EMDR Flash Technique group were shown to be statistically significant when compared to the mhGAP group. However, no statistically significant difference in the DASS-21 Depression, Stress, Impact of Event Scale-Revised Hyperarousal WHOQOL-BREF General Health, Physical, Social Relationships, and Environment component scores was reported between the two groups. The present study’s findings clearly demonstrate that the EMDR Flash technique, when applied to persons involved in traffic accidents, is successful in improving anxiety, intrusion, avoidance, total traumatic stress, and mental quality of life symptoms for at least 1 month. We believe that these findings will improve the reliability and applicability of the EMDR Flash Technique, which was tested for the first time in a clinical randomized-controlled trial (RCT).

## Introduction

Traumatic experiences that impact a person emotionally, behaviorally, and physiologically are difficult to live with and make regular living circumstances challenging (Raja et al., 2015). Despite the fact that exposure to traumatic life events varies, a 2019 study indicated that 70% of the population had experienced a traumatic incident at least once in their lives (2019). Various therapeutic intervention approaches for such a prevalent illness have been developed and implemented in clinical practice (Shapiro, 1989; Schouten et al., 2015). Individual interventions, on the other hand, are a key component of these strategies (Schouten et al., 2015). Due to variables such as financial constraints and a lack of qualified and licensed workers, only a small proportion of trauma victims have access to these procedures in this context (Davis and Maul, 2015). As a result, it is obvious that effective procedures that can be applied to more than one person in need are required.

Eye movement desensitization and reprocessing (EMDR) is a psychotherapy method developed by Shapiro (1989) for the treatment of trauma and associated disorders (Shapiro, 1989). According to the EMDR model, faulty and/or insufficient coding or processing of the traumatic experience may result in psychopathology owing to a lack of adaptive integration into memory (Hase et al., 2017). As a result, bilateral eye movements, also known as EMDR bilateral stimulation (BLS), indicate that it is successful in encoding and processing these unpleasant memories and related cognitions *via* distinct procedural stages to review and re-encode memory with auditory or tactile inputs (Hase et al., 2017). A great number of distinctive research have proved the usefulness of EMDR, and over time, approaches that propose its application to more than one individual have been presented (Gonzalez-Vazquez et al., 2018; Oztanriover et al., 2019; Doğan et al., 2021). EMDR Flash Technique is a relatively recent individual and group intervention that allows EMDR to be applied to a technical group at the same time (Manfield). Although the Flash Technique originated in the context of EMDR, there are publications suggesting that it should be considered as an independent trauma-related intervention that can presently be used outside of EMDR therapy (Brouwers et al., 2021; Manfield et al., 2021). Its effectiveness has already been proven in a small number of case studies and case series (Manfield et al., 2017; Yaşar et al., 2021). This limited literature indicates that the EMDR Flash technical intervention reduces trauma-related stress symptoms (Manfield et al., 2021; Yaşar et al., 2021). These reports analyze the EMDR Flash Technique before and after administration; however, there is no comparison with any other intervention strategy (Manfield et al., 2017, 2021; Wong, 2019; Yaşar et al., 2021). A randomized-controlled trial (RCT) of this novel approach is expected to disclose its effectiveness more clearly in clinical group.

Considering all this evidence, the aim of this study was to determine the efficacy of the EMDR Flash Technique in persons who had experienced traumatic events. In addition, utilizing an RCT design, we anticipated that participants in the EMDR Flash Technique group would report substantial improvements in measures of traumatic stress symptoms, anxiety, depression, stress, and quality of life compared to the mhGAP stress management module application group.

## Materials and Methods

In this randomized-controlled trial, the EMDR Flash Technique and mhGAP were evaluated using pre- and post-administration assessments and a one-month follow-up.

### Sample

**Figure 1:**
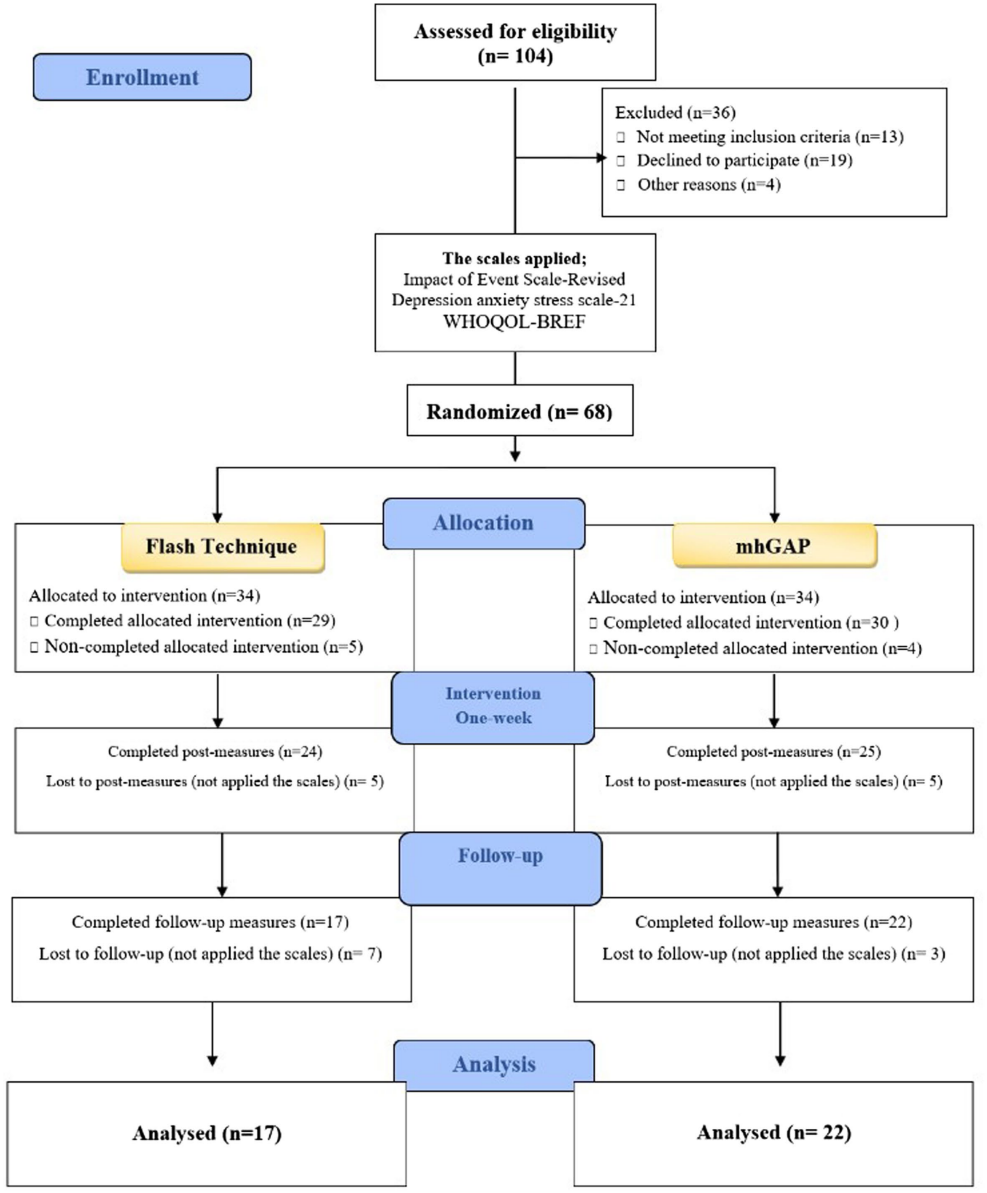
Flowchart of participants through each stage of the study.

One hundred and four people who were involved in a traffic accident filled out a Google Form to apply to the study. Due to the pandemic-related circumstances, the volunteers were interviewed by phone, and 13 people who did not fit the requirements to be included in the study, 19 participants who did not want to be included in the study, and 4 persons who were omitted due to technical problems (inability to contact the participant, lack of technical prerequisites for the application, etc.) were excluded. As a consequence, the remaining 68 individuals were randomized using the “research randomizer” tool, and it was determined to use the EMDR Flash Technique on 34 people and the mhGAP on 34 people. The study’s flow chart is presented in Figure 1.

The inclusion criteria of the study are as: (a) aging between 18 and 65, (b) having had a traffic accident between 6 months and 10 years ago, (c) not having organic mental complaints as a result of the traffic accident, d. having a score of <7 on the Adverse Childhood Experiences Questionnaire (Gunduz et al., 2018), (e) not having a psychiatric illness such as schizophrenia or bipolar disorder, (f) not having severe head traumas, (g) possessing the technological capabilities to implement the application, and (h) volunteering to participate in the study.

### Study Design

The study was carried out between February and October 2021 under the supervision of the Behavioral Sciences Institute. To reach people who had been in car accidents, an announcement was posted on social media, the research was detailed, and volunteers were asked to give their contact information using Google Form. The volunteers were interviewed and were thoroughly informed about the study, the inclusion requirements were assessed, and a Google Form information comprising data collecting tools was delivered to the participants who fulfilled the inclusion criteria. Following that, the subjects were randomly assigned to groups and the EMDR Flash Technique and mhGAP were applied to them online. Participants eligible for the applications were randomized to be 20–24 subjects. In other words, randomization was applied to three groups in total for the study. In both groups, the application was made for three consecutive days. Those who were unable to finish their applications at this stage were ruled out of the study. Participants were requested to complete out forms including data collection tools 1 week and 1 month after completing the applications. Participants who did not complete the forms were barred from participating in the study. Following appropriate processing, the obtained data were subjected to statistical analysis.

Before the study, power analysis was performed and when the α error: 0.05, power: 0.80, and effect size were calculated as 0.4, the sample size was found to be 18 for each group.

### Implementation

#### EMDR Flash Technique

The EMDR Flash Technique Group Protocol was employed in this study, and session lengths ranged from 60 to 75 min. Each group application was completed in three sessions. A 15-min introduction session was conducted at the first group session. At this stage, traumatic incidents were not discussed; instead, the group’s togetherness was the focus. The 15-min application was then presented. At this stage, EMDR therapy was presented briefly, and the technical specifics of the Flash Technique are introduced. This section requested the importance of the positive moment, as little contact with the traumatic memory as possible, the belief that it will be beneficial with some neurocognitive mechanisms, the requirement of being close to the screen to follow the blinks in the online application and taking a sitting position in order to do medicine. The participants were then asked to identify five troubling scenes connected to the traffic accident they witnessed and to rate the subjective units of distress intensity they felt immediately when focusing on each of them on a scale of zero to 10 (SUD score). After that, they were asked to recall a happy moment, dream, or image that brought them comfort when they thought about it. Examples were given to assist them to recall such a memory and a few people who were able to do so were encouraged to briefly share their happy recollections with others until everyone certainly had recalled at least one scene. The instructions were then explained, and repeated attempts were made to guarantee compliance. Flash Technique Desensitization was used from this stage until the last 10 min of the second and third sessions. In the desensitization phase, 5 bilateral stimulation sets and then, a triple blink (FLASH) application was performed six times in a row in accordance with the protocol used by Yaşar et al. (2021) in their study on traumatic stress, and the change in memory was questioned at short intervals, and then, this six-fold application was repeated. Requested to go on to other memories until the final part of the last session continued, when the memory with zero SUD value was over. Those who had all five memories reset were asked to recall another unpleasant aspect of the experience, if possible. If the memory they were working on was not reset, they were just instructed to keep working. The group sessions were concluded with comments in the final 10 min of the Third Group session.

#### mhGAP Stress Management Module

mhGAP (known as Improving Mental Health Training for Primary Care Residents in Turkey) is a WHO capacity-building, training/implementation program aimed at improving mental health services in primary healthcare settings. Stress management and associated disorders are covered as part of this training program. The mhGAP training program for primary care family physicians in Turkey has been carried out in collaboration with the Ministry of Health and the WHO Turkey office. One of the first-line strategies in the management of stress and related disorders is described as “Psychoeducation for stress and associated disorders” in this module. The aim of this training is to understand the origin of the individual’s stress symptoms and to make sense of the symptom cluster that emerges as a result of the stressor event. As a consequence, the tension created by the individual’s symptoms is also alleviated. In our study, our researcher, who is also one of the module’s trainers, administered recommended psychoeducation in the stress-related disorders module to the control group. The implementation of the mhGAP protocol in this study was applied in session lengths ranging from 55 to 65 min. Each group application was completed in three sessions.

### Ethics

All participants who agreed to enroll for the study were informed online and their consent was obtained. The study only included individuals who provided their consent. All stages of the study and data protection were carried out in line with the Helsinki Declaration, and the approval was obtained from the Clinical Research Ethics Committee of Marmara University Faculty of Medicine (26.07.2019/09.2019.707).

### Data Collection Tools

#### The Socio-Demographic Data Form

The socio-demographic data form was developed by the researchers in line with the literature and study objectives. Participant’s demographic data such as age, sex, educational level, and profession were inquired.

#### The Depression-Anxiety-Stress Scale-21

The Depression-Anxiety-Stress Scale-21 (DASS-21) was used in the study to determine the depression, anxiety, and stress symptoms of the participants. The scale has 21 items (Lovibond and Lovibond, 1995). Each question is evaluated on a four-point Likert-type scoring and it has seven items each for depression, anxiety, and stress. The score that can be obtained from the scale for each subdimension changes between 0 and 21. The Turkish validity and reliability study of the scale were conducted by Saricam (2018).

#### The Impact of Event Scale

The Impact of Event Scale (IES-R) was applied to measure the amount of effect of persons from an event in the study. It was developed by Weis and Marmara in 1997 (Weiss, 2007). It is a self-report five-point Likert-type scale and includes 22 items. It evaluates the level of exposure to events in three different fields as “Intrusion,” “Avoidance,” and “Hyperarousal.” The score that can be obtained from the scale varies between 0 and 88. The Turkish validity and reliability studies of the scale were carried out (Çorapçıoğlu et al., 2006).

#### The World Health Organization Quality of Life Instrument

The World Health Organization Quality of Life Instrument (WHOQOL-BREF-TR) was created by the WHO to assess the practitioner’s quality of life. The Turkish version of the scale has 27 items. The scale assesses the General Health, Physical, Psychological, Social Relationships, and Environment aspects of quality of life. An increase in the scale score depicts an improvement in one’s quality of life. The Turkish validity and reliability studies of the scale were conducted by Fidaner et al. (1999).

#### Adverse Childhood Experience Turkish Form

Adverse Childhood Experience Turkish Form (ACE-TR) was developed by Permanente in 1997 to inquire whether a person’s life prior to the age of 18 included domestic emotional violence, physical violence, sexual violence, abuse, emotional and physical neglect, and unpleasant childhood events such as divorce and being interrogated. It is a self-report scale consisting of 10 items. The Turkish validity and reliability studies of the scale were conducted by Gunduz et al. (2018).

### Statistical Analysis

SPSS 22 package program was used to perform statistical analyses on study data. Descriptive data were presented with frequency and percentage for categorical variables and mean and standard deviation for continuous variables. The Pearson Chi-square test for categorical variables and parametric assumptions for continuous variables were used to compare the pre-application variables of the two groups, followed by the Student’s *T*-test. The repeated measures analysis of variance was used to see whether EMDR Flash Technique and mhGAP procedures alone resulted in significant changes in the dependent variables and if there was a difference in changes between the two groups. In cases where the Mauchly test revealed that the sphericity assumption had been violated, the Greenhouse–Geisser adjustment was used, and the corrected results were reported. The effect size was determined using *η*^2^. A value of *p* < 0.05 was considered statistically significant.

## Results

The analysis of the study was carried out with a total of 39 participants, including 17 EMDR Flash Technique and 22 mhGAP who completed the study (Figure 1). The participants in the study had a mean age of 36.20 (11.41) years, with 82.1% (*n* = 32) being female. Table 1 compares socio-demographic and traffic accident-related ratings between the pre-intervention EMDR Flash Technique and mhGAP groups. As a result, there was no significant difference between the two groups when socio-demographic and traffic accident-related ratings were compared (*p* > 0.05).

**Table 1:** Baseline characteristics of mhGAP and flash technique participants.

	mhGAP (*n* = 22)	EMDR Flash Technique (*n* = 17)	*p*-value
Age; year, Mean (SD)	34.22 (11.00)	38.76 (11.76)	0.240
Gender (Female); *n* (%)	18 (81.8%)	14 (%82.4)	0.966
Marital Status (Single); *n* (%)	7 (31.8%)	6 (35.3%)	0.819
Education (University); *n* (%)	20 (90.9%)	14 (82.4%)	0.428
Time after the accident; month, Mean (SD)	36.00 (33.77)	29.88 (36.73)	0.592
Death in accident (Yes); *n* (%)	2 (9.1%)	2 (11.8%)	0.785
Physical injury in accident (Yes); *n* (%)	8 (36.4%)	9 (25.9%)	0.301
Hospitalization after the accident (Yes); *n* (%)	6 (27.3%)	6 (35.3%)	0.590
Physical injury to other individuals in the accident (Yes); *n* (%)	4 (18.2%)	4 (23.5%)	0.682
Psychological assistance due to accident (Yes); *n* (%)	4 (18.2%)	4 (23.5%)	0.682
Pre-accident mental assistance (Yes); *n* (%)	3 (13.6%)	3 (17.6%)	0.731
Adverse Childhood Experiences; year, Mean (SD)	1.63 (1.55)	2.64 (2.17)	0.100

mhGAP, Mental Health Gap Action Program; SD, Standard deviation.

There was no statistical difference between the two study groups pre-intervention in DASS-21 Anxiety (*p* = 0.704), Depression (*p* = 0.717), Stress (*p* = 0.355) subscale scores, Impact of Event Scale-Revised Intrusion (*p* = 0.482), Avoidance (*p* = 0.349), Hyperarousal (*p* = 0.657), and total (*p* = 0.925) scores, and WHOQOL-BREF General Health (*p* = 0.761), Physical (*p* = 0.124), Psychological (*p* = 0.710), Social Relationships (*p* = 0.432), and Environment (*p* = 0.268) component scores.

Table 2 compares DASS-21 Anxiety, Depression, and Stress subscale scores, Impact of Event Scale-Revised Intrusion, Avoidance, Hyperarousal, and total scores, and WHOQOL-BREF General Health, Physical, Psychological, Social Relationships, and Environment component scores in the EMDR Flash Technique and mhGAP groups before, 1 week, and 1 month after the study, within and between groups. As a result, there was a statistically significant difference in the DASS-21 Depression [*F*_(2–42)_ = 4.388, *p* = 0.019, *η*^2^ = 0.173], Stress [*F*_(2–42)_ = 4.117, *p* = 0.023, *η*^2^ = 0.164], Impact of Event Scale-Revised Intrusion [*F*_(2–42)_ = 12.621, *p* < 0.001, *η*^2^ = 0.375], Avoidance [*F*_(1–25)_ = 11.738, *p* < 0.001, *η*^2^ = 0.359], Hyperarousal [*F*_(2–42)_ = 6.695, *p* = 0.003, *η*^2^ = 0.242], Total [*F*_(1–28)_ = 15.343, *p* < 0.001, *η*^2^ = 0.422], WHOQOL-BREF Physical [*F*_(2–32)_ = 5.198, *p* = 0.012, *η*^2^ = 0.257] subscale scores in the mhGAP group, while no statistically significant difference was found in the DASS-21 Anxiety [*F*_(2–42)_ = 2.228, *p* = 0.120, *η*^2^ = 0.096], General Health [*F*_(1–20)_ = 2.133, *p* = 156, *η*^2^ = 0.118], Psychological [*F*_(2–32)_ = 14.641, *p* = 0.807, *η*^2^ = 0.013], Social Relationships [*F*_(2–32)_ = 1.215, *p* = 0.310, *η*^2^ = 0.071], and Environment [*F*_(2–32)_ = 0.946, *p* = 0.399, *η*^2^ = 0.059] component scores.

**Table 2:** Descriptive statistics for outcome variables in the mhGAP and flash technique.

	Methods	Pre-measurement	Post-measurement	Follow-up Measurement	Between Time Effect Size (*η*^2^)	Between Groups Effect Size by the time (*η*^2^)
**DASS-21; Mean (SD)**
Anxiety	mhGAP	5.09 (3.58)	3.63 (2.57)	3.77 (2.99)	0.096	0.085^*^
	Flash Technique	5.47 (2.23)	1.82 (1.38)	2.00 (1.76)	0.710^*^	
Depression	mhGAP	6.04 (3.82)	4.50 (3.55)	4.50 (3.54)	0.173^*^	0.024
	Flash Technique	5.58 (3.93)	2.88 (2.52)	3.11 (3.03)	0.385^*^	
Stress	mhGAP	7.50 (3.29)	6.09 (2.54)	5.72 (3.25)	0.164^*^	0.019
	Flash Technique	6.58 (2.59)	4.23 (2.99)	3.82 (2.62)	0.416^*^	
**Impact of Events Scale-Revised; Mean (SD)**
Intrusion	mhGAP	12.04 (7.91)	7.77 (6.92)	7.13 (6.37)	0.375^*^	0.101^*^
	Flash Technique	10.47 (5.16)	2.52 (2.45)	2.23 (2.94)	0.803^*^	
Avoidance	mhGAP	10.54 (5.81)	7.31 (4.06)	6.36 (3.69)	0.359^*^	0.124^*^
	Flash Technique	12.41 (6.44)	4.88 (2.80)	5.05 (4.03)	0.678^*^	
Hyperarousal	mhGAP	9.59 (5.75)	6.81 (3.88)	6.13 (4.76)	0.242^*^	0.071
	Flash Technique	8.82 (4.68)	3.35 (2.84)	2.58 (2.59)	0.708^*^	
Total	mhGAP	32.18 (16.57)	21.90 (12.32)	19.63 (13.01)	0.422^*^	0.147^*^
	Flash Technique	31.70 (14.03)	10.64 (7.16)	9.88 (8.63)	0.824^*^	
**WHOQOL-BREF; Mean (SD)**
General Health	mhGAP	53.67 (19.14)	58.08 (19.23)	62.50 (20.25)	0.118	0.058
	Flash Technique	55.83 (20.52)	70.83 (11.24)	71.66 (12.01)	0.487^*^	
Physical	mhGAP	48.52 (7.99)	51.74 (8.38)	55.13 (4.11)	0.257^*^	0.024
	Flash Technique	53.80 (10.83)	55.71 (7.49)	56.66 (8.41)	0.035	
Psychological	mhGAP	56.86 (10.81)	57.10 (7.62)	58.57 (11.73)	0.013	0.106^*^
	Flash Technique	55.55 (8.57)	64.72 (4.94)	65.27 (6.03)	0.495^*^	
Social relationships	mhGAP	62.25 (25.53)	63.23 (25.35)	67.15 (21.54)	0.071	0.012
	Flash Technique	56.11 (16.50)	61.11 (13.60)	62.77 (16.32)	0.126	
Environment	mhGAP	63.23 (14.12)	65.63 (13.32)	66.74 (13.45)	0.059	0.007
	Flash Technique	68.33 (10.94)	72.70 (9.55)	73.54 (9.29)	0.206^*^	

mhGAP: Mental Health Gap Action Program, SD: Standard deviation, DASS-21: Depression-Anxiety-Stress Scale-21, WHOQOL-BREF: World Health Organization Quality of Life Scale Abbreviated Version.

* *p* < 0.05.

There was a statistically significant difference in the DASS-21 Anxiety [*F*_(2–32)_ = 39.089, *p* < 0.001, *η*^2^ = 0.710], Depression [*F*_(2–32)_ = 9.996, *p* < 0.001, *η*^2^ = 0.385], Stress [*F*_(2–32)_ = 11.381, *p* < 0.001, *η*^2^ = 0.416], Impact of Event Scale-Revised Intrusion (F_(2–32)_ = 65.301, *p* < 0.001, *η*^2^ = 0.803), Avoidance [*F*_(2–32)_ = 33.685, *p* < 0.001, *η*^2^ = 0.678], Hyperarousal [*F*_(1–21)_ = 38.808, *p* < 0.001, *η*^2^ = 0.708], Total [*F*_(2–32)_ = 74.893, *p* < 0.001, *η*^2^ = 0.824], WHOQOL-BREF General Health [*F*_(1–16)_ = 13.265, *p* = 0.001, *η*^2^ = 0.487], Psychological [*F*_(2–28)_ = 11.541, *p* < 0.001, *η*^2^ = 0.495], and Environment [*F*_(2–28)_ = 3.634, *p* = 0.040, *η*^2^ = 0.206] subscale scores in the EMDR Flash Technique group, while no statistically significant difference was found in the Physical [*F*_(2–32)_ = 0.501, *p* = 0.611, *η*^2^ = 0.035] vs. Social relationships [*F*_(2–28)_ = 2.015, *p* = 0.152, *η*^2^ = 0.126] component scores.

**Figure 2:**
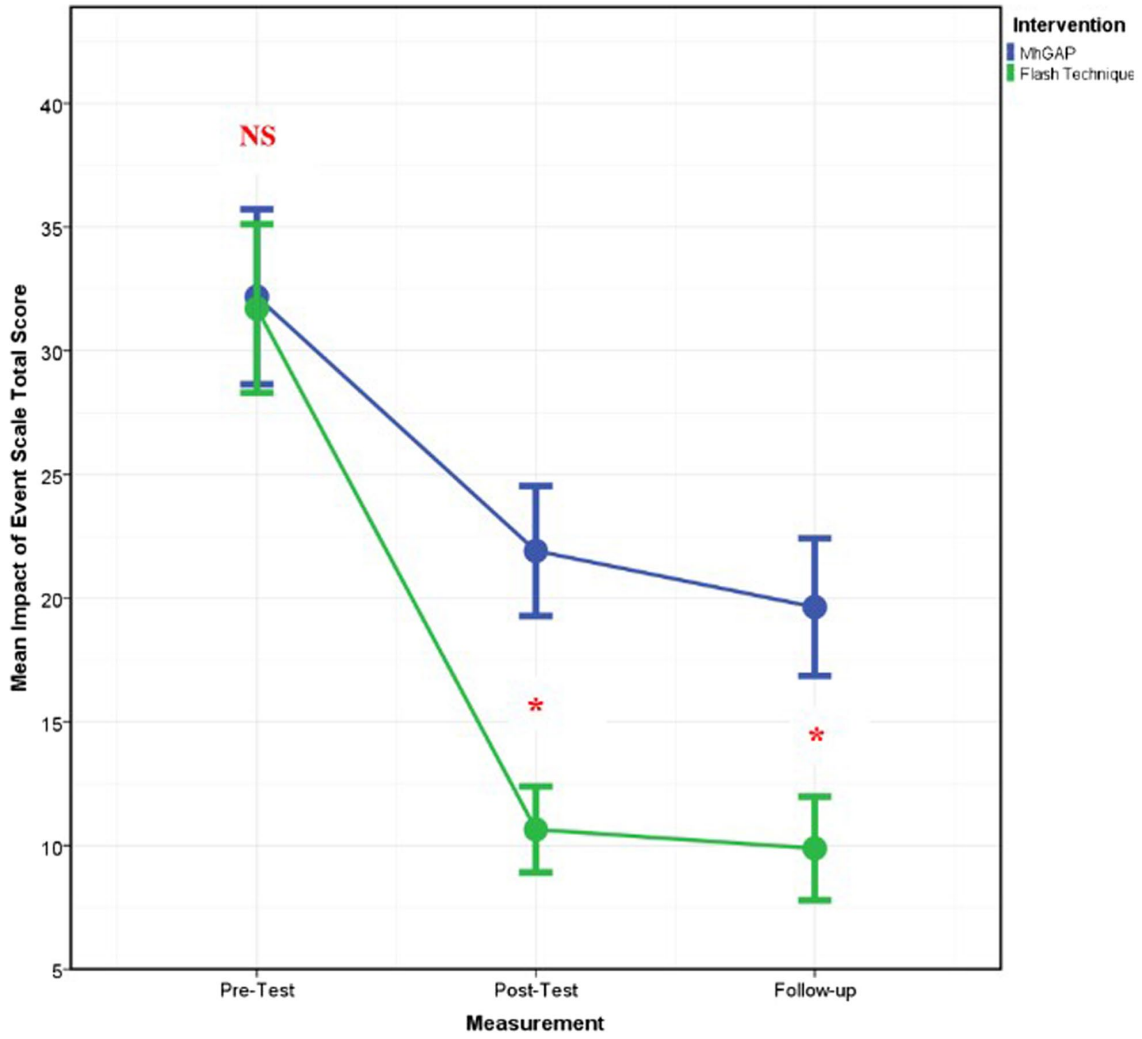
Graph of change of IES-R total score between two applications. *: statistically significant.

The change in DASS-21 Anxiety [*F*_(2–74)_ = 3.421, *p* = 0.038, *η*^2^ = 0.085], Impact of Event Scale-Revised Intrusion [*F*_(2–74)_ = 4.142, *p* = 0.020, *η*^2^ = 0.101], Avoidance [*F*_(2–74)_ = 5.220, *p* = 0.008, *η*^2^ = 0.124], Total (*F*_(2–74)_ = 6.363, *p* = 0.003, *η*^2^ = 0.147, Figure 2), and WHOQOL-BREF Psychological [*F*_(2–60)_ = 3.540, *p* = 0.035, *η*^2^ = 0.106] scores of the EMDR Flash Technique group was determined to be statistically significantly different compared to the mhGAP group. However, no statically significant difference was observed in the DASS-21 Depression [*F*_(2–74)_ = 3.609, *p* = 0.403, *η*^2^ = 0.024], Stress [*F*_(2–74)_ = 0.732, *p* = 0.484, *η*^2^ = 0.019], Impact of Event Scale-Revised Hyperarousal [*F*_(2–74)_ = 2.846, *p* = 0.064, *η*^2^ = 0.071], WHOQOL-BREF General Health [*F*_(2–60)_ = 1.855, *p* = 0.165, *η*^2^ = 0.058], Physical [*F*_(2–58)_ = 0.703, *p* = 0.499, *η*^2^ = 0.024], Social relationships [*F*_(2–60)_ = 0.351, *p* = 705, *η*^2^ = 0.012], and Environment [*F*_(2–60)_ = 0.198, *p* = 821, *η*^2^ = 0.007] component scores between the two groups.

## Discussion

The present research aims to examine the efficiency of the EMDR Flash Technique application for anxiety, depression, stress, trauma symptoms, and the quality of life in a sample affected by traffic accidents. According to the findings of the research, the EMDR Flash Technique application, which is based on the assumption that a given discomfort may be reduced by effectively reconstructing traumatic stress symptoms using the adaptive information processing model, has been determined to be effective in alleviating traumatic stress symptoms. The current results have explicitly demonstrated that the EMDR Flash technique application in the individuals emotionally affected by traffic injuries is effective in healing the individuals’ symptoms including anxiety, intrusion, avoidance, total traumatic stress, and the quality of mental state. The present research is the first clinical randomized control study to compare the effectiveness of the EMDR Flash Technique application with the mhGAP module, a structured application, on the assessment and management of stress-related conditions. We believe that these results will contribute to the reliability and applicability of the EMDR Flash Technique.

The primary result of this study was that traumatic stress symptoms were significantly reduced more in the EMDR Flash Technique group than in the mhGAP group. Depending on the measurements conducted, there were significant differences between the participants’ scores for traumatic stress in pre-treatment and post-treatment periods and during a one-month follow-up. These results obtained affirm the results of the articles indicating that the EMDR Flash Technique reduces traumatic stress symptoms (Shebini, 2019; Manfield et al., 2021; Wong, 2021; Yaşar et al., 2021) and feature a superiority regarding the greater effectiveness that it showed in comparing a group having the similar traumatic story of socio-demographic and ACE scores such as marital status with a randomized group. It is possible to state that the findings of this study will contribute to the reliability of the EMDR Flash Technique. Furthermore, this specific application features some particular aspects. First, compared to EMDR Therapy solely, the application of the EMDR Flash Technique protocol way more practical as it does not require questioning and/or thinking about the details related to traumatic cases. Moreover, compared to the standard EMDR Therapy, clients shortly contact to traumatic memories for several times throughout the therapy and thus, the risk of dissociation is reduced for the participants and their level of comfort is elevated. Less contact to traumatic cases and a relatively easier application can be among the advantages of the EMDR Flash Technique for the group therapy format. This allows the method to be applied to a plurality of individuals. Therefore, it can be stated that the present study well proves the reliability and safety of the EMDR Flash Technique.

Another significant finding of the present research is that the EMDR Flash Technique application is more effective in reducing anxiety than the mhGAP group. Furthermore, as an important aspect of the study, no significant difference was observed between the two groups while the levels of depression and stress were decreasing. Given their effectiveness in comparing anxiety levels, the mhGAP application can be said to have an effect on anxiety reduction. When examining these three results in conjunction, it can be seen that the selection of the mhGAP management as the control group preferable to demonstrate the effectiveness of the EMDR Flash Technique. In this context, the effectiveness of the EMDR Flash Technique was underscored again. Furthermore, it was pointed out that the EMDR Flash Technique was more effective in the quality of mental status with the present study. Noting that the present study focuses on the last 1 month to assess the quality of life, it can be concluded that further long-term research is required for more objective results (Fidaner et al., 1999).

Such an outstanding effectiveness of the EMDR Flash Technique can be understood with a variety of mechanisms. Manfield (2021) has suggested that the Technique enables traumas to be processes effectively by firstly being subjected to a traumatic memory for an instant, subsequently preventing the activation of the amygdala by inhibiting conscious thinking over the trauma while activating the prefrontal cortex. Accordingly, during the processing period, the Flash Technique aims to prioritize memory while excluding details and discomfort from conscious awareness (Manfield et al., 2021). Besides the hypotheses on the need for the existence of a contradictory experience to reconsolidate the trauma, it can be seen that the effect mechanism of the EMDR Flash Technique is quite robust considering its ability to process both positive and negative memories simultaneously (Ecker et al., 2012). On the other hand, Adaptive Information Processing is activated during the application of EMDR Flash Technique, which explains the logic of trauma processing in EMDR Therapy (Shapiro and Forrest, 2001). According to this model, a client becomes an observer against the trauma resulting from a negative memory and his prefrontal cortex is activated by being processed during the application, the activation of his traumatic memories is inhibited and thus, the client is enabled to have a more positive perspective against the trauma with a reduced level of affection and emotional memory (Manfield et al., 2021). On the other hand, another effect mechanism of EMDR Flash Technique can be through Working Memory Theory. Accordingly, while the working memory of the human being performs two tasks at the same time, the discomfort of the traumatic memory decreases and this change is consolidated (de Jongh et al., 2013; Schwabe et al., 2014). In addition, another possible mechanism may be the subliminal exposure theory. Accordingly, it is thought that the activation of the amygdala prevents the processing of the traumatic memory, but the continuation of the processing is ensured by the dual tasking inhibiting the activation of the amygdala (Brouwers et al., 2021; Manfield et al., 2021; Siegel et al., 2022). In conclusion, considering rapid effectiveness of the EMDR Flash Technique, it should be noted that it may have an effect mechanism which is quite novel and has unexplained aspects.

The findings of the present research are required to be interpreted within some limitations. First, it should be noted that the implementations within the study were conducted on the participants using online methods due to the COVID-19 pandemic. This can be interpreted as both a limitation and an indication of the effectiveness of the implementations. As another limitation, the follow-up period was kept short to reduce the rate of drop-out. It is preferable to plan longer periods of follow-up for prospective studies. Another limitation of the study could be the absence of a shortlist control group. Differences in traffic accidents experienced by study participants were attempted to be restricted using various criteria, as shown in Table 1; however, proper homogeneity may not be provided. Moreover, it should be noted that the participants are likely to be manipulated as the scales employed in the study are the type of self-reports. Finally, the absence of the pre-registration of the clinical trial protocol can be considered as another limitation.

## Conclusion

In conclusion, the present study, which is the first clinical randomized control study to examine the EMDR Flash Technique, has indicated that the EMDR Flash Technique is effective and safe in reducing traumatic stress symptoms. Furthermore, it has been found out that the application of EMDR Flash Technique is more effective in alleviating traumatic stress symptoms compared to the mhGAP method. According to these findings, the study urges the application of the EMDR Flash Technique for the groups suffering from negative memories. It will be advantageous if prospective randomized control studies have longer periods of follow-up and focus on possible effect mechanisms.

## Data Availability Statement

The raw data supporting the conclusions of this article will be made available by the authors upon appropriate request.

## Ethics Statement

All participants who agreed to participate were informed online and their consent was obtained. The study only included individuals who provided their consent. All stages of the study and data protection were carried out in line with the Helsinki Declaration, and the approval was obtained from the Clinical Research Ethics Committee of Marmara University Faculty of Medicine (26.07.2019/09.2019.707).

## Author Contributions

AY, İG, EK, ÖK, and EU contributed to conception and design of the study and wrote sections of the manuscript. AY, İG, AT, and EU organized the database. İG, EU, and AT performed the statistical analysis. AY wrote the first draft of the manuscript. All authors contributed to manuscript revision, read, and approved the submitted version.

## Conflict of Interest

The authors declare that the research was conducted in the absence of any commercial or financial relationships that could be construed as a potential conflict of interest.

## Publisher’s Note

All claims expressed in this article are solely those of the authors and do not necessarily represent those of their affiliated organizations, or those of the publisher, the editors and the reviewers. Any product that may be evaluated in this article, or claim that may be made by its manufacturer, is not guaranteed or endorsed by the publisher.
